# Dysregulated bile acid metabolism drives lipid peroxidation and ferroptosis in NAFLD: therapeutic potential for traditional Chinese medicine

**DOI:** 10.3389/fphar.2025.1669805

**Published:** 2025-09-18

**Authors:** Jing Liu, Fuxing Li, Qianru Zeng, Wenxiao Hu, Le Yang, Shengping Luo, Dingxiang Li, Yihui Deng

**Affiliations:** ^1^ School of Integrated Chinese and Western Medicine, Hunan University of Chinese Medicine, Changsha, China; ^2^ Hunan Province Key Laboratory of Cerebrovascular Disease Prevention and Treatment of Integrated Traditional Chinese and Western Medicine, Hunan University of Chinese Medicine, Changsha, China; ^3^ Pulmonary Medicine-Respiratory and Critical Care Medicine, Ningxiang Traditional Chinese Medicine Hospital, Changsha, China; ^4^ School of Traditional Chinese Medicine, Hunan University of Chinese Medicine, Changsha, China; ^5^ The First Affiliated Hospital of Hunan University of Chinese Medicine, Changsha, China

**Keywords:** non-alcoholic fatty liver disease, bile acids, lipid peroxidation, ferroptosis, traditional Chinese medicine

## Abstract

Non-alcoholic fatty liver disease (NAFLD), characterized by abnormal lipid accumulation in hepatocytes, is prevalent in conditions such as type 2 diabetes mellitus and obesity, which are associated with dysregulated glucose and lipid metabolism. Bile acids (BAs) are critical regulators of lipid and glucose homeostasis. Emerging research suggests that disturbances in BA metabolism not only exacerbate metabolic imbalance but also promote ferroptosis via lipid peroxidation. This review differs by systematically linking BA regulation, ferroptosis, and TCM, highlighting the multi-component and multi-target advantages of TCM in preventing and treating NAFLD. We summarize the mechanisms by which BAs regulate hepatic lipid synthesis and oxidation, and how lipid peroxidation connects to ferroptosis through glutathione/glutathione disulfide (GSH/GSSG) and reactive oxygen species (ROS). Finally, we review studies on TCM modulation of BA metabolism and ferroptosis to improve lipid peroxidation and metabolic disorders, providing timely insights into innovative therapeutic strategies for NAFLD.

## Highlights

Insulin resistance boosts lipogenesis, reduces fatty acid oxidation, and triggers NAFLD.

Bile acids participate in hepatic steatosis, hepatocyte ballooning, and hepatic fibrosis.

Bile acids regulate lipid peroxidation and ferroptosis via FXR activation and signaling.

TCM prevents fat accumulation, fibrosis, stress, and inflammation, and regulates bile acid and microbiota levels.

## 1 Introduction

Nonalcoholic fatty liver disease (NAFLD) is a group of diseases caused by excessive lipid accumulation in the liver, often associated with metabolic disorders such as obesity, diabetes, dyslipidemia, and hypertension (Chalasani et al., 2018). Its pathological progression is closely related to abnormalities in lipid metabolism, oxidative stress, and cell death. The occurrence of NAFLD is often associated with insulin resistance and lipid metabolism disorders, such as type 2 diabetes mellitus (T2DM), hyperlipidemia, obesity, and other endocrine diseases. Insulin resistance not only aggravates lipid deposition but also significantly increases the risk of cardiovascular disease, kidney disease, and other metabolic complications (Yang et al., 2016a; Kim et al., 2024). Epidemiological studies show that about 70% of T2DM patients also have NAFLD, and the risk of insulin resistance and T2DM is two to three times higher in NAFLD patients compared to healthy individuals (Younossi et al., 2024). Therefore, studying the interaction mechanism between dyslipidemia and NAFLD is of great significance for improving patient prognosis, reducing complications, and enhancing clinical treatment outcomes.

In recent years, ferroptosis—a form of programmed cell death dependent on iron ions and lipid peroxidation—has garnered significant attention in the pathogenesis of NAFLD (Li et al., 2020c). Studies have shown that bile acid metabolism disorders disrupt lipid metabolic balance and promote ferroptosis by inducing lipid peroxidation, thereby participating in the pathological mechanisms of NAFLD (Quintero et al., 2014). Although numerous studies have explored the relationships between bile acid metabolism, lipid peroxidation, and ferroptosis, many questions remain unresolved. Despite numerous studies on the relationship between bile acid metabolism, lipid peroxidation, and ferroptosis, many questions remain. Existing reviews often treat these processes separately or focus on just one or two aspects. Additionally, while Traditional Chinese Medicine (TCM) is increasingly recognized for its multi-target therapeutic potential, few studies have focused on how TCM interventions modulate bile acid metabolism, improve lipid peroxidation, and regulate ferroptosis in the treatment of NAFLD.

**FIGURE 1:**
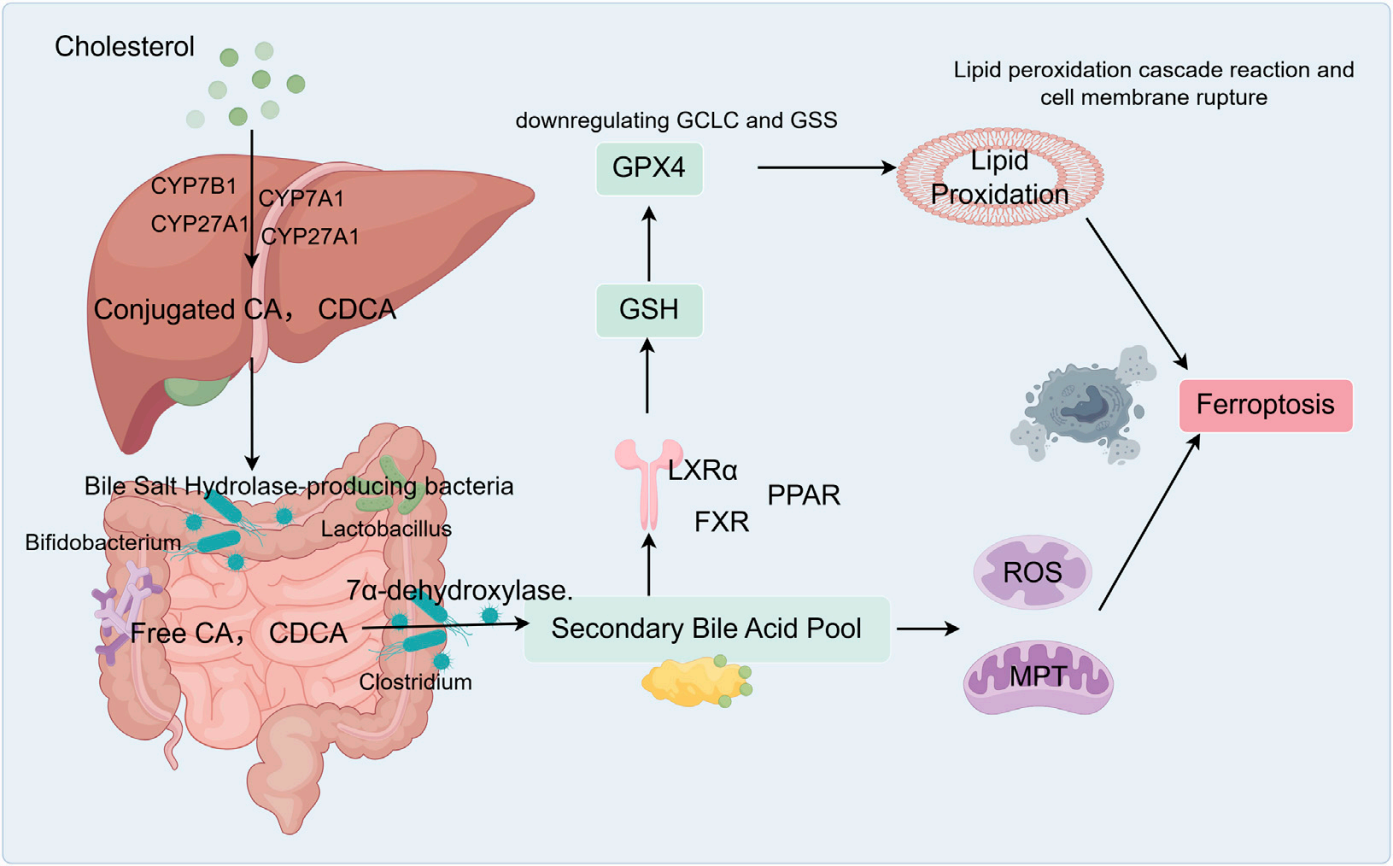
Graphical abstract: Bile acids participate in ferroptosis and influence lipid metabolism in NAFLD. The liver synthesizes primary bile acids, such as cholic acid (CA) and chenodeoxycholic acid (CDCA), from cholesterol. These primary bile acids conjugate with glycine or taurine to form conjugated bile acids. In the small and large intestines, anaerobic bacteria produce bile salt hydrolase (BSH), which deconjugates these primary bile acids, generating free CA and CDCA. Free CA enters the colon, where specific *Clostridium* species convert it via 7 α-dehydroxylase into the secondary bile acid deoxycholic acid (DCA). Free CDCA is similarly converted into lithocholic acid (LCA) by these bacteria. Hydrophobic bile acids (DCA, CDCA, and LCA) activate FXR, which inhibits genes responsible for glutathione synthesis (GCLC and GSS), thereby reducing GSH production. DCA directly damages mitochondria, triggering the release of reactive oxygen species (ROS) that attack membrane phospholipids, leading to the accumulation of lipid peroxides. Together with ROS, the GPX4 pathway ultimately induces ferroptosis.

This review first outlines how bile acids regulate lipid synthesis and oxidation, and how these processes intersect with lipid peroxidation and ferroptosis in NAFLD. We then examine key signaling pathways, particularly those involving glutathione/glutathione disulfide (GSH/GSSG), reactive oxygen species (ROS), and ferroptosis-related lipid metabolism. Next, we summarize evidence on Traditional Chinese Medicine (TCM) interventions and their potential to modulate bile acid metabolism, attenuate lipid peroxidation, and regulate ferroptosis. Finally, we discuss translational implications and future research directions (Figure 1).

## 2 Glucose and lipid metabolism in non-alcoholic fatty liver disease

### 2.1 Epidemiology

NAFLD is a group of diseases characterized by excessive lipid accumulation in the liver, with its pathological progression closely linked to abnormalities in lipid metabolism, oxidative stress, and cell death (Chalasani et al., 2018). NAFLD has become the leading cause of chronic liver disease worldwide, with a global adult prevalence of 29.9%–34.9%. The prevalence in men (39.7%) is significantly higher than in women (25.6%). Notably, the prevalence in overweight/obese individuals reaches 51.6%, while 14.4% in individuals with normal weight (Riazi et al., 2022). Multiple factors drive the pathogenesis of NAFLD, including abnormalities in lipid metabolism and insulin resistance. These factors contribute to fat accumulation, chronic inflammation, and gut microbiota dysbiosis, further aggravating hepatic cell damage and metabolic abnormalities (Buzzetti et al., 2016; Marra and Svegliati-Baroni, 2018; Byrne and Targher, 2020). In NAFLD patients, hepatic lipid accumulation is closely associated with metabolic disorders such as obesity, T2DM, hypertriglyceridemia, and hypertension, as well as cardiovascular diseases (Quek et al., 2023; Younossi et al., 2024). Among these, insulin resistance is a key factor contributing to lipid metabolism disorders. Therefore, NAFLD and T2DM often coexist and influence each other.

Diabetes has become a global public health issue and is the eighth leading cause of death and disability worldwide (GBD, 2019 Diseases and Injuries Collaborators, 2020). According to the latest GBD 2021 study published in The Lancet, 529 million people were living with diabetes worldwide in 2021, and 96.0% of these cases were T2DM (GBD, 2021 Diabetes Collaborators, 2023). Both diabetes and NAFLD are chronic metabolic diseases, and if left uncontrolled for an extended period, they can lead to dysfunction, disability, and various severe complications. It is estimated that 60%–70% of T2DM patients have NAFLD, and 20%–30% of these patients may progress to non-alcoholic steatohepatitis (NASH). Additionally, the risk of NAFLD progressing to cirrhosis in T2DM patients is 2–3 times higher than in non-diabetic individuals (Younossi et al., 2024). In conclusion, NAFLD and T2DM are closely linked global public health challenges that influence each other. Studying their pathogenesis and new therapeutic approaches is essential for reducing incidence, delaying progression, and improving patient prognosis.

### 2.2 Physiopathological mechanism

The liver is the body’s primary metabolic organ, responsible for various functions, including synthesizing, breaking down, and transporting lipids. It maintains the balance of lipids in the body by synthesizing and breaking down fatty acids, cholesterol, and triglycerides (Nguyen et al., 2008). The physiological functions of lipid metabolism include providing energy, synthesizing cell membranes, and producing hormones and vitamins. Normal lipid metabolism supplies energy to cells and participates in critical physiological processes such as cell signal transduction and immune responses (Prentki and Madiraju, 2008; Dukewich et al., 2025). Dietary lipids are primarily absorbed in the intestines and transported through the blood to adipose tissue and the liver. In NAFLD, triglycerides are hydrolyzed into diglycerides, monoglycerides, and glycerol, releasing fatty acids in the process. On the one hand, the increased fatty acid load is delivered to the liver, where it is cleared by enzymes such as fatty acid translocase, fatty acid transport proteins, and caveolin, and then incorporated into lipid droplets (Wilson et al., 2016; Enooku et al., 2020; Geng et al., 2021). On the other hand, fatty acids are oxidized in the mitochondria, peroxisomes, and microsomes, accompanied by an increase in the production of ROS (Geng et al., 2021; Moreno-Fernandez et al., n.d.). Lipid metabolism disorders are primarily characterized by abnormal fat accumulation in the liver, resulting in hepatocyte injury and dysfunction. The pathological mechanisms include insulin resistance, oxidative stress, and inflammatory responses (Bugianesi et al., 2005; Hirsova et al., 2016). The pathological features of fatty liver mainly include abnormal accumulation of fat in hepatocytes, liver inflammation, and fibrosis, which may eventually lead to severe consequences such as cirrhosis and liver cancer (Lee et al., 2015; Yang et al., 2022). In NAFLD, the liver’s role in lipid metabolism disorders—particularly the contributions of insulin resistance and oxidative stress—is central to the disease’s pathology. These factors lead to abnormal fat accumulation and exacerbate liver damage through multiple pathways.

Insulin resistance is a key factor contributing to lipid metabolism disorders. Under conditions of insulin resistance, liver cells exhibit reduced responsiveness to insulin, resulting in an inability to effectively inhibit fatty acid synthesis and the regular promotion of fatty acid oxidation. This metabolic abnormality leads to the accumulation of fatty acids in the liver, ultimately causing fatty liver disease. Chronic insulin resistance and fat accumulation further exacerbate the liver’s burden, laying the groundwork for the development of other metabolic disorders [25,26]. Oxidative stress is another key factor in lipid metabolism disorders. The liver is an organ with active oxidative metabolism in the body. Oxidative stress arises from the excessive production of ROS and free radicals, leading to lipid peroxidation and liver cell damage (Sumida et al., 2013). Oxidative stress exacerbates hepatocyte damage, increases the infiltration of F4/80-positive macrophages, reduces their activation, and promotes the expression of pro-inflammatory cytokines and genes, thereby further exacerbating hepatic inflammatory responses (Ji et al., 2019). As the disease progresses, the persistent presence of oxidative stress can lead to endothelial autophagy, endoplasmic reticulum stress, and ultimately hepatic fibrosis, potentially resulting in liver failure (Ruart et al., 2019; Abulikemu et al., 2023). Therefore, understanding the role of lipid metabolism in liver diseases provides new perspectives and potential therapeutic targets for the prevention and treatment of related diseases.

### 2.3 Interactions between glucose metabolism and lipid metabolism

Globally, 60%–70% of patients with T2DM also have NAFLD, and 20%–30% of these patients may progress to nonalcoholic steatohepatitis (NASH) (Younossi et al., 2024). The underlying mechanisms of this progression primarily include insulin resistance, disrupted hepatic fatty acid metabolism, oxidative stress, and enhanced inflammatory responses. Insulin resistance is a key pathogenic feature of metabolic syndrome, and NAFLD is closely associated with reduced systemic insulin sensitivity and increased insulin resistance in the liver and adipose tissue. On one hand, insulin promotes glycolysis, improves glucose utilization, inhibits hepatic glycogenolysis and gluconeogenesis, and suppresses hepatic glucose output (Saltiel and Kahn, 2001; Titchenell et al., 2017). On the other hand, insulin promotes cholesterol and fatty acid synthesis in the liver and enhances the re-esterification of fatty acids into triglycerides in adipocytes and the liver (Uehara et al., 2023). Under physiological conditions, insulin regulates hepatic glucose production by modulating lipolysis in adipose tissue, thereby reducing the influx of fatty acids into the liver (Rebrin et al., 1996). In NAFLD patients with insulin resistance, glucose undergoes glycolysis in the liver, ultimately being converted into fatty acids, which are then esterified into triglycerides. Triglycerides are the primary lipid form in the livers of patients with NAFLD (Donnelly et al., 2005; Heeren and Scheja, 2021). Insulin resistance leads to increased hepatic gluconeogenesis, enhanced glucose and fat production, and disrupted glucose and lipid metabolism, resulting in excessive production of free fatty acids (FFAs). FFAs enter hepatocytes and are converted into triglycerides, promoting hepatic lipid deposition and increasing the risk of NAFLD (Biddinger et al., 2008; Vatner et al., 2015)。

The primary transcription factors involved in insulin-mediated fat synthesis are Upstream Stimulator Factor (USF) (Wang and Sul, 1997), sterol regulatory element-binding protein-1c (SREBP-1c) (Raghow et al., 2008), carbohydrate response element-binding protein (ChREBP) (Postic et al., 2007), and liver X receptor alpha (LXRα). SREBP family genes are believed to regulate genes involved in controlling cholesterol homeostasis and *de novo* fatty acid synthesis (Raghow et al., 2008). SREBP-1a transactivates genes involved in lipogenesis and cholesteryl ester synthesis (Foretz et al., 1999). In contrast, SREBP-2 regulates genes involved in cholesterol biosynthesis and metabolism. ChREBP is a key glucose-activated transcription factor that regulates approximately 50% of lipogenesis in the liver (Iizuka et al., 2004; Herman and Samuel, 2016). The primary function of ChREBP is to regulate fructose metabolism and participate in the expression of genes involved in carbohydrate transport, glycolysis, and *de novo* lipogenesis (Iizuka et al., 2004; Jeong et al., 2011). The interaction between ChREBP and SREBP-1c coordinates postprandial glycolysis and lipogenesis in the liver (Linden et al., 2018). These results indicate that SREBP-1c is involved in insulin-induced activation of lipid synthesis genes, while ChREBP mediates glucose-induced activation of glycolysis and lipid synthesis genes. LXR is a ligand-activated nuclear receptor, and the expression of SREBP-1c and ChREBP is regulated by LXR, which controls the expression of genes involved in glycolysis and lipid synthesis, thereby playing a crucial role in the transcriptional control of lipid metabolism (Cha and Repa, 2007; Wang and Tontonoz, 2018). In summary, insulin resistance can lead to hepatic lipid accumulation by increasing FFA transport to the liver, enhancing *de novo* lipogenesis, and reducing hepatic fatty acid oxidation, thereby driving disease progression.

## 3 Biological functions of bile acids

### 3.1 Synthesis and secretion of bile acids

Bile acids (BAs) primarily consist of primary and secondary bile acids. Primary bile acids, such as cholic acid (CA) and chenodeoxycholic acid (CDCA), are synthesized from cholesterol in the liver. In contrast, secondary bile acids, such as lithocholic acid (LCA) and deoxycholic acid (DCA), are produced through the metabolism of intestinal microbiota. They participate in metabolic processes such as fat digestion and absorption, cholesterol metabolism, and immune regulation (Wahlström et al., 2016; Guzior and Quinn, 2021). 95% of bile acids are reabsorbed in the ileum and transported back to the liver to recycle and regulate *de novo* synthesis. The remaining 5% reaches the colon, where it is metabolized by the gut microbiota and excreted in feces (Fiorucci et al., 2021). In the classical pathway, the hydroxylation of cholesterol at the C-7 position is catalyzed by cholesterol 7α-hydroxylase (CYP7A1), which is exclusively distributed in the liver and serves as the rate-limiting enzyme for bile acid synthesis. Then, 7α-hydroxy cholesterol is further modified by sterol 27-hydroxylase (CYP27A1), ultimately forming CA and CDCA (Russell and Setchell, 1992). CYP27A1 effectively catalyzes the first rate-limiting step in the alternative pathway, while 7α-hydroxysteroid 7α-hydroxylase (CYP7B1) participates in subsequent 7α-hydroxylation, responsible for the majority of CDCA synthesis (Mihalik et al., 2002). Approximately 95% of bile acid reabsorption depends on the apical sodium-dependent bile salt transporter (ASBT) in the terminal ileum (Dawson et al., 2003; Li et al., 2020b). Bile acids bind to intrahepatic bile acid-binding protein (I-BABP) within cells. They are secreted into the portal venous circulation via the OSTα/β heterodimer on the basolateral membrane (Rao et al., 2008). After entering the portal vein, bile acids are taken up by hepatocytes via the organic anion transporting polypeptide (OATP) and the sodium-taurate cotransporter polypeptide (NTCP) on the basolateral membrane (Meier and Stieger, 2002; Suga et al., 2017).

BAs are a class of cholesterol metabolites, and their synthesis and excretion constitute the primary pathways for cholesterol catabolism. The metabolic regulatory roles of BAs in lipid and glucose metabolism, as well as insulin sensitivity, are associated with the expression of the FXR and TGR5 receptors (Zhang et al., 2006). Bile acids participate in the pathophysiological processes of NAFL and NASH through multiple mechanisms. FXR and TGR5 are widely expressed in tissues and organs, including the liver, intestines, white adipose tissue, and heart. Several distinct mechanisms in the liver and intestine regulate bile acid homeostasis by activating the FXR and TGR5 receptors. FXR inhibits the transcription of the CYP7A1 gene by suppressing the expression of hepatic nuclear factor 1α and hepatic nuclear factor 4α, which in turn inhibits SREBP1c and reduces lipid synthesis in the liver (Goodwin et al., 2000; Lu et al., 2000). In the intestine, FXR stimulates the expression of fibroblast growth factor 15 (FGF15) or FGF19, which in turn inhibits hepatic CYP7A1 gene transcription, thereby directly or indirectly suppressing SREBP1c and aiding in the inhibition of hepatic lipid synthesis (Inagaki et al., 2005; Di Ciaula et al., 2022). Hepatic gluconeogenesis can be suppressed by activating FXR, which inhibits the expression of several key transcription factors mediated by SHP (Yamagata et al., 2004). Activated FXR in the intestine induces FGF15/19, which dephosphorylates and inactivates the cAMP response element-binding protein, further inhibiting the expression of gluconeogenesis-related genes (Potthoff et al., 2011). In summary, FXR induces SHP expression in the liver, thereby inhibiting CYP7A1 expression. In the intestine, FXR increases the circulating levels of FGF19/FGF15, thereby reducing the expression of CYP7A1 and CYP8B1 and inhibiting bile acid synthesis (Shapiro et al., 2018).

### 3.2 Bile acids and NAFLD

The liver-gut axis is the bidirectional communication system between the liver and the gut. Nutrients, microbial antigens, metabolites, and bile acids regulate the metabolism and immune responses of the gut and liver, thereby mutually shaping the structure and function of the microbiota (Bruneau et al., 2021; Tilg et al., 2022). The function of the gut-liver axis depends on the interaction between the liver, gut, and microbiota. Gut microbiota influence liver metabolism and immune function, and the disruption of gut barrier integrity and host-microbiota interactions are essential factors in the development of NAFLD (Li et al., 2022b; Forlano et al., 2024). In the hepaticointestinal axis, bile acids act as crucial signaling molecules, regulating bile secretion, lipid metabolism, and glucose metabolism in the liver. Studies have found that circulating bile acid levels are associated with histopathological and genetic determinants of the progression from NAFLD to NASH (Nimer et al., 2021). Therefore, bile acids have become an essential target for preventing and treating NAFLD. Specifically, NASH livers exhibit elevated levels of TCA and TDCA in bile acid metabolomics, while CA and glycocholic acid (GDCA) levels are reduced (Lake et al., 2013). In NAFLD subjects, glycocholate, taurocholate, and glycochenodeoxycholate levels are significantly elevated (Kalhan et al., 2011). The ratio of bile acids to taurocholate and secondary bile acids to primary bile acids is negatively correlated with NAFLD activity scores. When the secondary bile acid ratio is high, an increased level of conjugated bile acids may be associated with a higher risk of significant fibrosis (Puri et al., 2018). Bile acid accumulation in the liver may exacerbate liver disease, with CDCA and glycine-conjugated bile acids related to higher macrovesicular steatosis scores, elevated serum ALT levels, and a larger quantified fibrosis area (Jia et al., 2014). Administration of CA or ursodeoxycholic acid (UDCA) is associated with reduced hepatic triglyceride levels and complete reversal of histological steatosis (Quintero et al., 2014). Changes in bile acids are closely related to hepatic steatosis, hepatocyte ballooning, and liver fibrosis.

Bile acids not only directly act on the liver to regulate hepatic steatosis but may also prevent the progression of NAFLD by activating the FXR receptor. FXR plays a crucial role in mediating communication between the host and the gut microbiota, particularly through regulating the enterohepatic circulation of bile acids (Zhang et al., 2016). The potency of bile acids in activating FXR is ranked as follows: CDCA > DCA > LCA > CA (Wang et al., 1999). Compared to NAFLD patients, NASH patients exhibit reduced levels of FXR, SHP, and NTCP proteins, and FXR plays a protective role in the progression from NAFLD to NASH (Aguilar-Olivos et al., 2015). Alterations in the gut microbiota result in increased levels of tauroconjugated bile acids (TCBAs) in both the gut and the liver. TCBA activates thermogenic adipose tissue and regulates blood glucose through FXR and TGR5 receptor-mediated signaling (Münzker et al., 2022). TUDCA inhibits the expression of FXR and fatty acid transport protein 5 (FATP5), thereby reducing fatty acid absorption and hepatic lipid accumulation, enhancing intestinal barrier function, and promoting the growth of *Allobaculum* and *Bifidobacterium* (Wang et al., 2024b). FXR-deficient mice are resistant to obesity induced by a high-fat diet, and transferring their microbiota to germ-free wild-type mice reduces obesity and improves glucose tolerance, indicating that the gut microbiota promotes weight gain and hepatic steatosis in an FXR-dependent manner (Parséus et al., 2017). The mechanism by which intestinal FXR activation shapes the gut microbiota to activate TGR5/GLP-1 signaling to improve hepatic glucose and insulin sensitivity and increase adipose tissue browning (Pathak et al., 2018). In summary, bile acids play a crucial role in the early prevention of NAFLD through pathways such as FXR regulation and the gut-liver axis. They may also serve as potential therapeutic targets for NAFLD and its complications.

## 4 Lipid peroxidation and ferroptosis

### 4.1 The relationship between lipid peroxidation and ferroptosis

Lipid peroxidation is the most significant type of oxidative free radical damage in biological systems, comprising a free radical chain reaction with three distinct stages: initiation, propagation, and termination (Valgimigli, 2023). The process of lipid peroxidation typically begins with the generation of free radicals. Cellular organelles, such as mitochondria and the endoplasmic reticulum, produce ROS during metabolic processes, which stimulate the generation of free radicals through various pathways. When free radicals come into contact with lipids, they can cause the unsaturated bonds in lipids to break, generating a lipid free radical (Lai and Piette, 1978; Greene et al., 2017). The generated lipid free radicals can react with oxygen to form lipid peroxy radicals, which attack surrounding lipid molecules and expand the lipid peroxidation chain reaction (Porter, 2013). Antioxidants can react with lipid free radicals, stabilize them, and prevent further oxidative reactions (Jones and Saso, 2007). Alternatively, lipid peroxides (LOOH) can react with other free radicals to form inactive products, halting the oxidative process (Saraev and Pratt, 2024). Polyunsaturated fatty acids (PUFAs) are generally considered the primary substrates for lipid peroxidation. The abundance of PUFAs determines the extent of available lipid peroxidation sites, thereby influencing susceptibility to ferroptosis (Yang et al., 2016b). The final products of lipid peroxidation, including hydrogen peroxide (H_2_O_2_), peroxides, and malondialdehyde (MDA), can disrupt cellular membrane structure and contribute to pathological processes such as inflammatory responses and cell death (Spitz et al., 1990; Ayala et al., 2014). Lipid peroxides generated by lipid peroxidation cause damage to the structure and function of cellular membranes, thereby triggering cell death, particularly playing a key role in iron-dependent death processes.

Ferroptosis is a non-apoptotic form of regulated cell death driven by iron-dependent lipid peroxidation, primarily occurring within cells. It is characterized by reduced mitochondrial volume, increased double membrane density, and reduced or absent mitochondrial cristae, while the cell membrane remains intact and the nucleus maintains normal size (Dixon et al., 2012). Intracellular glutathione (GSH) depletion, decreased activity of glutathione peroxidase 4 (GPX4), and the inability of lipid peroxides to be metabolized through the reductive reaction catalyzed by GPX4 lead to Fe^2+^ oxidizing lipids and generating a large amount of reactive oxygen species (ROS), thereby promoting ferroptosis (Chen et al., 2022). At the molecular mechanism level, the initiation of ferroptosis is closely related to three principal regulatory axes: regulation of the GSH/GPX4 pathway, regulation of iron metabolism, and regulation of pathways related to lipid metabolism (Li et al., 2020a). GPX4 converts GSH into oxidized glutathione (GSSG) and reduces cytotoxic LOOH to their corresponding alcohols (L-OH) (Liu et al., 2023). GSH, as a substrate for GPX4, also plays a key role in anti-ferroptosis. Changes in GSH metabolism ultimately lead to alterations in cellular sensitivity to ferroptosis (Friedmann Angeli et al., 2014). Therefore, inhibition of GPX4 activity can lead to the accumulation of lipid peroxides, which are markers of ferroptosis. The dynamic balance of the intracellular free iron pool plays a decisive role in ferroptosis sensitivity. Excess iron promotes lipid and ROS production through iron-dependent Fenton reactions and catalytic lipid peroxidation chain reactions, while also acting as a cofactor for lipoxygenases in the peroxidation modification of polyunsaturated fatty acids (Henning et al., 2022). Abnormal activation of lipid metabolic enzyme systems (such as ACSL4 and LOXs) promotes the synthesis of phospholipids containing polyunsaturated fatty acids, which are more susceptible to oxidative damage (Shah et al., 2018; Zhang et al., 2022).

### 4.2 The impact of ferroptosis on the NAFLD

Iron overload and lipid peroxidation are the primary characteristics of ferroptosis. Previous studies have reported that ferroptosis is associated with the pathogenesis of various diseases, such as tumors (Bai et al., 2019), kidney injury (Friedmann Angeli et al., 2014), encephalopathy (Hambright et al., 2017), and NAFLD (Li et al., 2020c). The liver is the primary regulatory organ for iron homeostasis, including iron absorption, utilization, storage, and secretion (Protchenko et al., 2021), promoting systemic regulation of iron homeostasis and storing excess iron in cases of iron overload (Gao et al., 2022). Iron overload is a crucial factor in NAFLD, and ferroptosis plays a role in its pathogenesis. A close relationship exists between the grading of hepatic steatosis and the expression of several genes involved in ferroptosis, including GSS, ACSL4, and ACSL3 (Dai et al., 2022). The ferroptosis inducer RSL3 reduces hepatic expression of GPX4, exacerbating hepatic steatosis and inflammation in NASH mouse models (J et al., 2020). In contrast, the ferroptosis inhibitor ferrostatin-1 (Fer1) significantly reduces lipid accumulation and markedly lowers hepatic triglyceride levels, alleviating inflammation, fibrosis, and liver damage in NASH mice (Li et al., 2020c). Iron-induced lipid peroxidation is a primary trigger of NAFLD, with GPX4 serving as a key regulator that mediates lipid peroxidation-induced ferroptosis. Hepatocyte lipid accumulation leads to endoplasmic reticulum stress and mitochondrial dysfunction and increases sensitivity to lipid peroxidation by altering membrane phospholipid composition. Under the synergistic effects of iron overload and lipotoxicity, GPX4 activity in hepatocytes is doubly inhibited: on one hand, iron ions weaken GPX4 substrate supply by consuming GSH (Kose et al., 2019). On the other hand, accumulated lipid peroxides directly deplete GPX4’s enzymatic activity reserves (Luo et al., 2023). This vicious cycle leads to the collapse of the hepatocyte antioxidant defense system, ultimately triggering irreversible ferroptosis.

**FIGURE 2:**
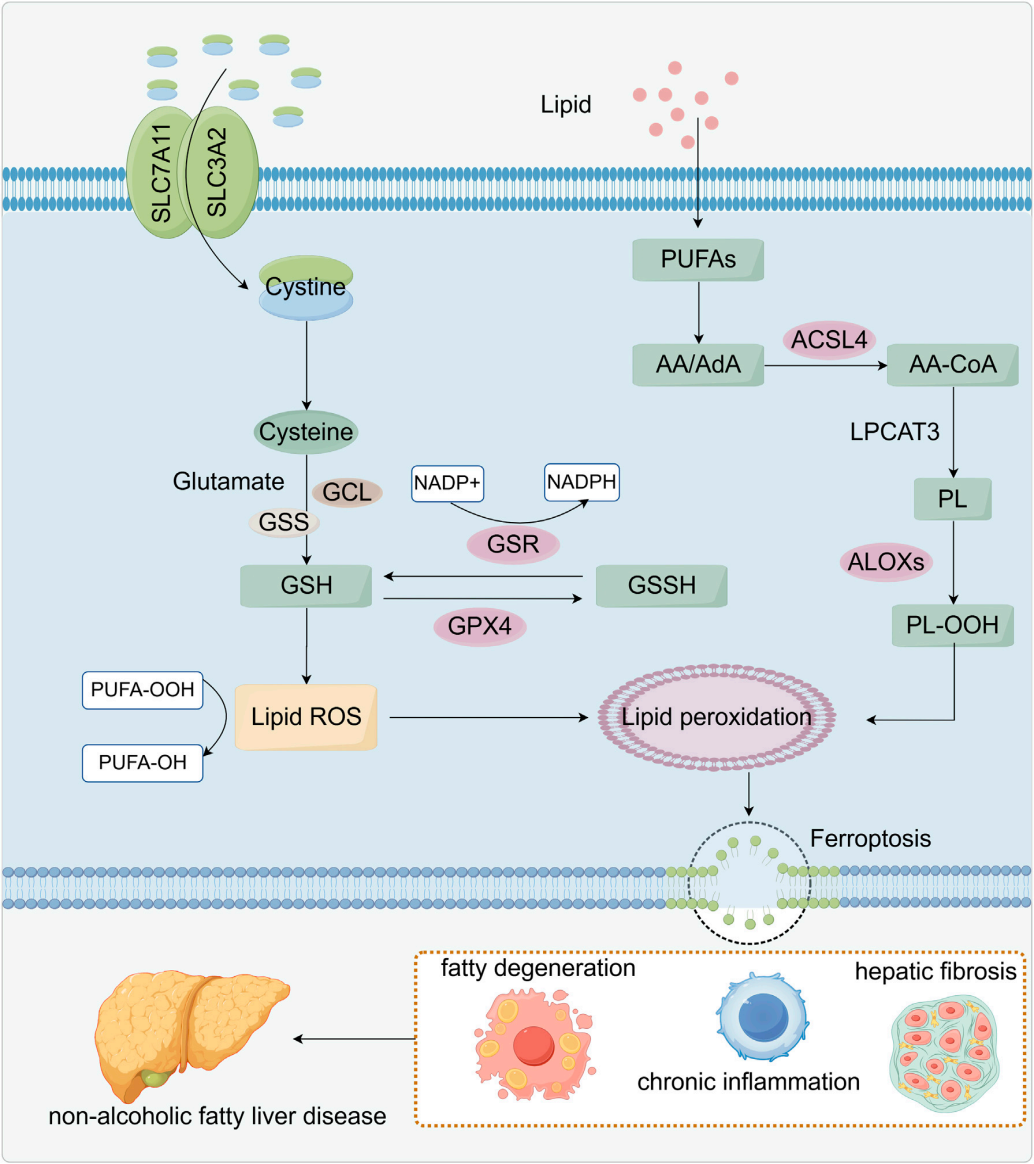
Relationship between lipid peroxidation and ferroptosis. SLC7A11 and SLC3A2 on the cell membrane co-transport cystine, which is reduced to cysteine and used with glutamate to synthesize glutathione (GSH). Glutathione reductase (GSR) uses NADPH to regenerate GSH from its oxidized form (GSSG), sustaining the antioxidant cycle. GSH also works through glutathione peroxidase 4 (GPX4) to eliminate lipid ROS and inhibit lipid peroxidation. Meanwhile, polyunsaturated fatty acids (PUFAs) are esterified by ACSL4 and LPCAT3 into phospholipids, which ALOX enzymes then oxidize to peroxidized phospholipids (PL-OOH), thereby fueling lipid peroxidation. When GSH is depleted or GPX4 is inhibited, lipid ROS accumulate, driving ferroptosis. These metabolic disturbances lead to steatosis, chronic inflammation, and hepatic fibrosis, ultimately promoting the development of NAFLD.

Macrophages in the liver recycle large amounts of free iron released by the iron-induced death of hepatocytes. Iron accumulation promotes their polarization toward a pro-inflammatory phenotype. At the same time, free iron also regulates the tricarboxylic acid cycle in macrophages, thereby further regulating the production of inflammatory cytokines (M et al., 2019). Iron overload promotes the production of ROS in T cells, leading to DNA damage and impairing T cell responsiveness, thereby accelerating the inflammatory pathological process (Wang et al., 2018). Therefore, the release of free iron from hepatic iron death significantly promotes the initiation of the inflammatory cascade. Clinical studies have reported hepatic iron overload in NAFLD and NASH patients, which is associated with increased severity and progression of NAFLD (Corradini et al., 2021). Serum ferritin levels help identify patients with NAFLD who are at risk for NASH and advanced fibrosis (Kowdley et al., 2012; Jung et al., 2019). In summary, hepatic steatosis, chronic inflammation, and hepatic fibrosis are the primary characteristics of NAFLD. Ferroptosis significantly promotes the progression of NASH/NAFLD by facilitating the development of hepatic steatosis, chronic inflammation, and hepatic fibrosis. Current iron death regulatory strategies targeting GPX4 demonstrate potential therapeutic value. Liproxstatin-1 inhibits GPX4-induced iron death (Fan et al., 2021, p. 4), while N-acetylcysteine alleviates mitochondrial oxidative damage and iron death by enhancing mitochondrial GSH activity and mitochondrial redox homeostasis (Li et al., 2022a). The harmful role of non-classical GPX4 subtypes in ferroptosis has been demonstrated, and selective targeting of non-classical GPX4 transcription variants (iGPX4) may represent a promising therapeutic strategy for MAFLD (Tong et al., 2022). FXR acts as a guardian against ferroptosis by upregulating the expression of anti-ferroptotic genes, thereby reducing lipid peroxidation (Tschuck et al., 2023). These findings provide a theoretical basis for developing NAFLD therapeutic drugs targeting the ferroptosis pathway (Figure 2).

## 5 Bile acid-mediated lipid peroxidation and ferroptosis

### 5.1 Bile acids and lipid peroxidation

#### 5.1.1 Bile acids and regulation of lipid metabolism

Bile acids, as end products of cholesterol metabolism, play a central role in lipid homeostasis. Through activation of the farnesoid X receptor (FXR), bile acids suppress sterol regulatory element-binding protein 1c (SREBP-1c), thereby reducing fatty acid and triglyceride synthesis (Cha and Repa, 2007; Wang and Tontonoz, 2018). They also enhance fatty acid oxidation, particularly through the activation of peroxisome proliferator-activated receptor alpha (PPARα). Hyodeoxycholic acid (HDCA) improves NAFLD via PPARα activation, inhibits abnormal nuclear–cytoplasmic shuttling, and reduces lipid accumulation and oxidative stress (Zhong et al., 2023). Clinical studies have revealed that HDCA levels are significantly reduced in individuals with obesity, diabetes, and NAFLD (Zheng et al., 2021). Obeticholic acid (OCA), a synthetic bile acid, activates the FXR to regulate bile acid and lipid metabolism (Clifford et al., 2021). However, microbiota-induced lipid peroxidation can impair its antifibrotic effects (Zhuge et al., 2022). Usodeoxycholic acid (UDCA) exerts protective effects against liver damage by antagonizing FXR, thereby regulating lipid absorption, reducing lipid accumulation, and mitigating oxidative stress (Mueller et al., 2015). Tauroursodeoxycholic acid (TUDCA), a taurine-conjugated form of UDCA, helps alleviate NAFLD by modulating gut microbiota and bile acid metabolism, while preventing oxidative stress and mitochondrial dysfunction (Wang et al., 2024b). Thus, bile acids influence both fatty acid synthesis and oxidation through multiple pathways, impacting lipid homeostasis and contributing to the development of metabolic disorders when dysregulated.

#### 5.1.2 Hydrophobic and hydrophilic bile acids in oxidative stress

Hydrophobic bile acids (BAs) such as cholic acid (CA) and deoxycholic acid (DCA) are closely associated with lipid peroxidation and hepatocellular injury. Lipid peroxidation, characterized by the accumulation of lipid peroxides, is a significant contributor to oxidative stress and mitochondrial dysfunction. Hydrophobic BAs stimulate ROS production through multiple mechanisms. First, BAs enhance oxidative phosphorylation in mitochondria, thereby increasing ROS output. In hepatic stellate cells, hydrophobic bile acids also induce epidermal growth factor receptor (EGFR) phosphorylation, leading to ROS production via NADPH oxidase activation (Sommerfeld et al., 2009). At the mitochondrial level, these BAs promote mitochondrial permeability transition (MPT) and cytochrome c release, which subsequently trigger apoptosis (Yerushalmi et al., 2001). Experimental studies have demonstrated that taurocholate sulfate induces ROS accumulation in a dose-dependent manner, thereby impairing ATP production and leading to apoptosis and necrosis (Booth et al., 2011). Glycochenodeoxycholic acid (GCDCA) induces mitochondrial permeability transition in a dose-dependent manner, leading to excessive ROS generation and the release of cytochrome c and apoptosis-inducing factor (AIF), ultimately contributing to apoptosis (Botla et al., 1995; Sokol et al., 2005). In contrast, hydrophilic bile salts such as ursodeoxycholic acid (UDCA) can mitigate BA-induced mitochondrial injury. UDCA decreases ROS production and Bax protein expression, stabilizes mitochondrial transmembrane potential, and inhibits cytochrome c release, thereby protecting hepatocytes from DCA-induced apoptosis (Botla et al., 1995; Rodrigues et al., 1998).

Bile acids influence lipid metabolism and oxidative stress via multiple pathways. Hydrophobic bile acids exacerbate oxidative damage and mitochondrial dysfunction, while hydrophilic bile acids offer protective effects. These dual roles highlight the therapeutic potential of targeting bile acid metabolism and modulating lipid peroxidation in NAFLD (Table 1).

**TABLE 1:** Summary of bile acid mechanisms in lipid peroxidation and liver disease.

Bile acid	Research type	Mechanisms	References
Hydrophobic bile acids	*In vitro*	Induces NADPH oxidase phosphorylation → ROS formation	Sommerfeld et al. (2009)
Hydrophobic bile acids	*In vitro*	Dose- and time-dependent ROS, mitochondrial permeability transition, cytochrome C release	Yerushalmi et al. (2001)
Taurocholate sulfate	*In vitro*	Dose-dependent ROS increase; impaired ATP production	Booth et al. (2011)
Glycochenodeoxycholic acid	*In vitro*	Induces the MPT in a dose-dependent manner, stimulates ROS generation, and releases cytochrome c and apoptosis-inducing factor	Botla et al. (1995), Sokol et al. (2005)
Ursodeoxycholic acid	*In vivo*	Inhibits the MPT, reduces DCA-induced ROS, Bax, and mitochondrial permeability transition	Botla et al. (1995), Rodrigues et al. (1998)
Cholic Acid and Muricholic Acid	*In vitro* and vivo	Reduces BA secretion and lipid absorption via mitochondria–peroxisome–ER tethering	Da Dalt et al. (2024)
Hyodeoxycholic acid	*In vivo* and clinical trials	Activates PPARα, enhancing FA oxidation and reducing lipid accumulation	Mueller et al. (2015), Zhong et al. (2023)
Obeticholic acid	*In vivo* and clinical trials	Activates FXR, reduces unsaturated FA, decreases lipid absorption	Clifford et al. (2021)
Chenodeoxycholic acid	*In vivo*	Reduces TG with PUFA, increases free PUFA and PC-PUFA	Zhuge et al. (2022)
Tauroursodeoxycholic acid	*In vivo*	Inhibits FXR and FATP5 → reduces FA absorption and accumulation	Wang et al. (2024b)

### 5.2 Bile acids and ferroptosis

Bile acid metabolism plays a key role in lipid metabolism and energy balance, while ferroptosis is a form of cell death triggered by iron-dependent lipid peroxidation. Both processes involve the accumulation of peroxides of polyunsaturated fatty acids, leading to interactions in their pathological mechanisms. On the one hand, bile acid metabolism plays a direct role in regulating ferroptosis through lipid peroxidation. Studies have shown that DCA upregulates the expression of hypoxia-inducible factor-2α (HIF-2α) and divalent metal transporter-1 (DMT1), leading to the accumulation of ferrous ions, which enhances lipid peroxidation within cells and promotes the onset of ferroptosis (Wang et al., 2024a). UDCA is a naturally occurring, low-toxicity, hydrophilic bile acid in the human body, converted from primary bile acids by intestinal microbiota. UDCA binds to the cystine transporter SLC7A11, inhibiting cystine uptake and impairing *de novo* GSH, which leads to ROS accumulation and mitochondrial oxidative damage (Xie et al., 2025). TCA reduces ferritin heavy chain 1 (FTH1) and ferroptosis-related protein levels, upregulates intracellular iron, reactive oxygen species, and lipid peroxidation levels, thereby exacerbating ferroptosis (Zeng et al., 2025). On the other hand, bile acids can regulate molecules associated with ferroptosis by activating the nuclear receptor FXR and its downstream signaling pathways. Bile acids activate FXR to inhibit ferroptosis, and FXR activation significantly reduces lipid peroxidation by upregulating ferroptosis gatekeepers GPX4, FSP1, PPARα, SCD1, and ACSL3, thereby acting as a guardian of ferroptosis (Tschuck et al., 2023). BAs can reverse Erastin-induced ferroptosis in gastric cancer, and BAs significantly increase the expression of glutathione synthase and GPX4 by activating FXR, thereby inhibiting ferroptosis sensitivity (Liu et al., 2024). FXR activation rescues lipid peroxidation, intracellular ROS, and Fe^2+^ accumulation, inhibits ferroptosis, and alleviates nephrotoxicity (Tang et al., 2023). Bile acids function as signaling molecules in regulating ferroptosis; however, current research on the regulation of hepatic ferroptosis by bile acids is limited, and further validation of their interaction mechanisms is needed in the future.

## 6 Traditional Chinese medicine intervention strategies

### 6.1 TCM regulation of bile acids in NAFLD

Currently, treatment options for NAFLD include lifestyle changes, fecal microbiota transplantation, surgery, and drug therapy. However, there are still no specific drugs for NAFLD, and no drugs have been approved for this condition. TCM, especially herbal medicine or herbal extracts, is receiving increasing attention due to its multi-component, multi-pathway, and multi-target characteristics, which give it unique advantages in preventing and treating NAFLD.

#### 6.1.1 Enhancing bile acid synthesis and excretion

Penthorum chinense Pursh induces an alternative pathway of chenodeoxycholic acid (CDCA) synthesis, activates FXR, promotes BA excretion, and reduces cholesterol levels, effectively ameliorating NAFLD in mouse models (Li et al., 2022d). Based on data mining, the bioactive components of Penthorum chinense Pursh bind to RXRA and FXR, significantly increasing the expression of RXRA, FXR, and bile salt export pump (BSEP) in L02 cells, while decreasing the expression of CYP7A1 (Li et al., 2022c). Isoquercetin activates alternative BA biosynthesis pathways and inhibits intestinal FXR-FGF15 signaling, which reduces cholesterol and triglyceride levels in the liver, improving NAFLD (Zhang et al., 2023). Nuciferine elevates conjugated BA and non-12OH BA levels, downregulates protein levels of FXR, FGF15, FGFR4, and ASBT, and upregulates protein levels of CYP7A1 and CYP27A1, decreases BSH-producing genus, 7α-dehydroxylation genus, and increases taurine metabolism-related genus (Sun et al., 2022). Diosgenin can regulate BAs metabolism through the liver FSR-shp and intestinal FSR-FGF15 pathways, especially in the CA, TCA, and treat NASH (Yan et al., 2023). Diosgenin can inhibit excessive weight gain in rats with NAFLD induced by a high-fat diet, reduce serum total cholesterol and triglyceride levels, and decrease liver fat accumulation. It also regulates bile acid metabolism, particularly lithocholic acid and ursodeoxycholic acid 3-sulfate (Zhou et al., 2022). Glycyrrhizin regulates BA metabolism, inhibits deoxycholic acid-induced NLRP3 inflammasome activation, and improves hepatic inflammation, steatosis, and fibrosis in NAFLD (Yan et al., 2018).

#### 6.1.2 Modulating gut microbiota and BSH-Producing bacteria

Sanye Tablet enhances taurine-conjugated bile acid levels in the liver and feces, upregulating enzymes involved in BA synthesis. It reduces lipid accumulation and suppresses BSH-producing bacteria, improving liver lipid metabolism and preventing hepatic steatosis in rodent models of NAFLD (Qi et al., 2025). Thyme polyphenol-rich extract reduces serum total BA levels and increases fecal BA levels, enhancing the relative abundance of *Lactobacillus* species, which positively influences bile acid metabolism and mitigates high-fat diet-induced NAFLD (Sheng et al., 2024). Ling-Gui-Zhu-Gan Decoction influences BA biosynthesis and PPAR signaling, modulates gut microbiota composition, and reduces hepatic steatosis in NAFLD mice (Chen et al., 2024). Hyperoside, a flavonol glycoside found in various herbs, exhibits antioxidant, hepatoprotective, and anti-inflammatory properties. It has been shown to increase the expression of liver FXR and LXRα, promote fatty acid oxidation, and enhance BA efflux from the liver. Additionally, it regulates hepatic *de novo* lipogenesis and BA metabolism, inhibits gut microbes associated with bile salt hydrolase (BSH) activity, and reduces cholesterol and triglyceride levels in NAFLD rats (Wang et al., 2021; Wang et al., 2025b). Epigallocatechin-3-gallate (EGCG) has been shown to exert beneficial effects on metabolic disorders and fatty liver disease. EGCG reduces BA reabsorption, decreases intestinal BA levels, and lowers lipid absorption, mitigating metabolic disorders and fatty liver disease induced by a high-fat diet (Huang et al., 2018; Ushiroda et al., 2019). Additionally, EGCG induces a hepatospecific decrease in CYP3A expression levels. This effect is attributed to alterations in the intestinal flora (Ikarashi et al., 2017). Further studies confirm that EGCG’s ability to lower intestinal BA levels and reduce lipid absorption plays a critical role in alleviating fatty liver and metabolic disorders (Naito et al., 2020).

In conclusion, these bioactive compounds and herbal formulations demonstrate promising therapeutic potential for managing NAFLD by regulating bile acid metabolism, enhancing liver function, and improving lipid homeostasis (Table 2).

**TABLE 2:** TCM regulation of bile acids in NAFLD.

Herb/Formulation	Targets	Mechanisms	Research types	References
Penthorum chinense Pursh	FXR, BSH-producing bacteria, TUDCA, TCDCA, CDCA	Regulates gut microbiota and bile acid metabolism, reduces cholesterol, and improves NAFLD	*In vivo*	Li et al. (2022d)
Isoquercetin	FXR, FGF15	Reduces Hepatic Cholesterol and Triglyceride in NAFLD Mice by Modulating Bile Acid Metabolism	*In vivo*	Zhang et al. (2023)
Diosgenin	CA, TCA, lithocholic acid, ursodeoxycholic acid 3-sulfate	Reduces serum total cholesterol and triglyceride levels, and Decreases liver fat accumulation	*In vivo*	Zhou et al. (2022), Yan et al. (2023)
Nuciferine	conjugated BA and non-12OH BA FXR CYP7A1 and CYP27A1	Regulates BA metabolism and modulates the gut microbiota	*In vivo*	Sun et al. (2022)
Glycyrrhizin	FXR, NLRP3 inflammasome	Improves hepatic steatosis, inflammation, and fibrosis	*In vivo*	Yan et al. (2018)
Hyperoside	FXR, LXRα, and BSH-producing bacteria	Reduces lipid accumulation and improves liver function	*In vivo*	Wang et al. (2021), Wang et al., (2025b)
Sanye Tablet	FXR, Bile acid synthesis enzymes, BSH-producing bacteria	Reduces hepatic steatosis and improves lipid metabolism	*In vivo*	Qi et al. (2025)
Thyme polyphenol-rich extract	*Lactobacillus*, fecal conjugated BA	Alleviates hepatic steatosis, modulates gut microbiota	*In vivo*	Sheng et al. (2024)
Ling-Gui-Zhu-Gan Decoction	CYP7A1/FXR, PPAR	Improves NAFLD by suppressing the growth of bile acid-producing bacteria and promoting the growth of short-chain fatty acid-producing bacteria	*In vivo*	Chen et al. (2024)
EGCG	LCA, TCA, CYP7A1, CYP27A1, HMG-CoA reductase, taurine	Reduces bile acid reabsorption, lowers intestinal bile acid levels, and reduces lipid absorption to alleviate NAFLD	*In vitro* and vivo	Ikarashi et al. (2017), Huang et al. (2018), Ushiroda et al. (2019), Naito et al. (2020)
Curcumin	CDCA, TCA, LCA	Regulates amino acids, TCA cycle, bile acids, and gut microbiota	Clinical trials	Chashmniam et al. (2019)

### 6.2 TCM regulation of lipid peroxidation and ferroptosis in NAFLD

Ferroptosis, a regulated form of cell death driven by lipid peroxidation, is increasingly recognized as a key mechanism in the progression of NAFLD. Several Chinese herbal medicines and their bioactive compounds have been shown to modulate lipid peroxidation and ferroptosis, offering new therapeutic strategies for NAFLD.

#### 6.2.1 Antioxidant defense and GPX4/GSH enhancement

Quercetin is widely recognized as an essential flavonoid with anti-inflammatory and antioxidant properties (Anand David et al., 2016). Quercetin increases GPX4 and GSH/GSSG ratios, inhibits ROS, lipid peroxides, and iron overload, and suppresses iron-induced cell death in hepatocytes and the liver, alleviating NAFLD (Jiang et al., 2022; Huang et al., 2023). It also inhibits inflammasome responses and the activation of the endoplasmic reticulum stress pathway, thereby improving lipid metabolism and alleviating inflammatory conditions associated with NAFLD (Porras et al., 2017; Zhu et al., 2018). EGCG can prevent GSH depletion, GPX4 inactivation, and lipid peroxidation by chelating iron ions, and is considered a novel inhibitor of ferroptosis (Kose et al., 2019). EGCG alleviates liver damage, lipid accumulation, oxidative stress, and hepatic steatosis, increases NRF2 and GPX4 expression in iron-overloaded mice, and enhances antioxidant capacity (Ding et al., 2023; Yang et al., 2023). It also improves intestinal microbiota dysbiosis in non-alcoholic NASH mice, thereby alleviating lipid accumulation and ferroptosis (Ning et al., 2020), indicating that EGCG is a potential inhibitor of ferroptosis. Dehydroabietic acid promotes the expression of GSH and GPX4, reduces ROS accumulation, reduces hepatic lipid peroxidation, inhibits hepatic ferroptosis, and improves NAFLD (Gao et al., 2021).

#### 6.2.2 Pathway-specific regulation of ferroptosis and lipid accumulation

Diosgenin significantly alleviates ferroptosis and ROS accumulation in HepG2 cells by regulating the ACSL4/LPCAT3/ALOX15 pathway, thereby mitigating NAFLD (Wang et al., 2025a). Diosgenin reduces lipid accumulation and steatosis, upregulates the expression of nuclear factor erythroid 2-related factor 2 and its downstream ferroptosis-related genes, and inhibits ferroptosis in the livers of rats with non-alcoholic fatty liver disease (Zhang et al., 2024). Through network pharmacology and *in vivo* validation, Chaihu Shugan Powder (CSP) has been shown to improve liver inflammation in a hepatic steatosis model. It inhibits hepatic fatty acid synthesis by inhibiting the TNFα/TNFR1 signaling pathway (Lei et al., 2022). CSP inhibits the AMPK-mTOR pathway, restores autophagy and ferroptosis markers such as GPX4 and Nrf-2, and alleviates non-alcoholic steatohepatitis by inhibiting autophagic ferroptosis (Liang et al., 2024). Ginkgolide B significantly improves oxidative damage and lipid peroxidation by blocking ferroptosis, with its mechanism of action related to Nrf2 activation (Yang et al., 2020). Flavonol rutin reduces hepatic ferritin expression and serum transferrin saturation, with its hepatoprotective effect associated with inhibiting hepatic ferroptosis (Hawula et al., 2021). Arbutin, a natural antioxidant, inhibits obesity-associated protein (FTO), which increases the m6A methylation level of SLC7A11, promotes the expression of SLC7A11, and ultimately inhibits iron death, slowing down the progression of NAFLD *in vivo* and *in vitro* (Jiang et al., 2023). Nuciferine Ameliorates Fatty Acid Accumulation and Iron Death via the PPARα Signalling Pathway, PPARα Inhibitors Block the Protective Effects of Nuc, Resulting in Excessive Accumulation of Iron Ions, Suggesting that Nuc May Be a Potential Drug for the Treatment of NAFLD (Qiu et al., 2024).

Their therapeutic effects primarily involve strengthening antioxidant defenses by enhancing GPX4 and GSH activity, reducing ROS accumulation, and limiting iron-induced lipid damage, thereby protecting hepatocytes. These mechanisms highlight TCM’s multifaceted potential for NAFLD therapy (Table 3).

**TABLE 3:** TCM regulation of lipid peroxidation and ferroptosis in NAFLD.

Herb/Formulation	Targets	Mechanisms	Research types	References
Quercetin	GPX4, ROS, GSH/GSSG ratios, and iron metabolism	Alleviates lipid peroxidation and oxidative stress	*In vivo*	Jiang et al. (2022), Huang et al. (2023)
EGCG	GSH, GPX4, Fe2+/Fe3+, mitochondrial reactive oxygen species, *Bacteroides*	Improves hepatic oxidative stress and lipid metabolism, and modulates the gut microbiota, thereby inhibiting ferroptosis, alleviating liver injury, and reducing lipid accumulation	*In vitro* and vivo	Kose et al. (2019), Ning et al. (2020), Ding et al. (2023), Yang et al. (2023)
Flavonol rutin	Transferrin receptor1 (TFR1), TFR2	decreases in liver ferritin protein levels and transferrin saturation	*In vivo*	Hawula et al. (2021)
Ginkgolide B	Nrf2, GPX4, TFR1, ferritin heavy chain-1	Activates the Nrf2 pathway, improves lipid deposition, and reduces ferroptosis caused by oxidative stress in NAFLD	*In vitro* and vivo	Yang et al. (2020)
Dehydroabietic acid	GSH, GPX4, ROS, ferroptosis suppressor protein 1 (FSP1)	Inhibits ferroptosis, ROS accumulation, and lipid peroxidation, and reduces HFD-induced NAFLD	*In vitro* and vivo	Gao et al. (2021)
Diosgenin	Nrf2, ACSL4, ROS, Fe2+	Reduces oxidative stress and inhibits of ferroptosis for the treatment of NAFLD	*In vitro* and vivo	Zhang et al. (2024), Wang et al. (2025a)
Chaihu Shugan Powder	GPX4, Nrf2, AMPK-mTOR	Alleviates liver inflammation, steatosis, and ferroptosis	*In vitro* and vivo	Lei et al. (2022), Liang et al. (2024)
Nuciferine	PPARα	Reduces fatty acid accumulation and ferroptosis, a potential NAFLD therapy	*In vitro* and vivo	Qiu et al. (2024)
Arbutin	FTO, SLC7A11	Alleviates NAFLD by acting on the FTO/SLC7A11 pathway to inhibit ferroptosis	*In vivo* and *in vitro*	Jiang et al. (2023)

### 6.3 Clinical evidence of TCM targeting bile acid metabolism in NAFLD

Clinical data indicate that curcumin modulates BA metabolism in NAFLD and can improve hepatic and metabolic indices. In a 24-week randomized controlled trial, curcumin reduced hepatic fat and remodeled BA signaling, suggesting a gut microbiota-dependent BA mechanism (He et al., 2024). Earlier metabolomics work in NAFLD has shown that curcumin shifts multiple pathways, including decreases in chenodeoxycholic acid, taurocholic acid, and lithocholic acid, alongside changes in amino acids and TCA cycle intermediates (Chashmniam et al., 2019). Supplementation with curcumin, combined with piperine, has been reported to significantly improve liver function, lower cholesterol levels, and increase total iron-binding capacity, thereby exerting therapeutic benefits in patients with NAFLD (Panahi et al., 2019). However, another clinical trial found that an 8-week course of curcumin with piperine did not significantly reduce serum pro-oxidant–antioxidant balance (PAB) values, suggesting that the administered dose may have been insufficient to achieve a measurable reduction in oxidative stress (Mirhafez et al., 2019). In addition, clinical evidence suggests that Fructus akebiae, when combined with ursodeoxycholic acid, has been shown to significantly alleviate clinical symptoms and improve serum biochemical parameters, including ALT, AST, triglycerides, and total cholesterol, in patients with NAFLD (Hongguang et al., 2019). Berberine ursodeoxycholate significantly reduced liver fat, improved liver enzymes and lipid profiles, promoted weight loss, and enhanced glycemic control, supporting its potential as a dual metabolic and hepatoprotective therapy for NAFLD/NASH despite mild gastrointestinal adverse events (Harrison et al., 2021). These findings highlight the potential of traditional Chinese medicine to modulate bile acid metabolism in the treatment of NAFLD, and suggest that combining bile acid-based therapies with traditional Chinese medicine may offer additional therapeutic benefits (Table 4).

**TABLE 4:** Clinical evidence of TCM targeting NAFLD.

Design	Intervention	BA endpoints	Main liver outcomes	Reference
RCT, n = 80, nonalcoholic simple fatty liver	Curcumin 500 mg/day, 24 weeks	↑ DCA, ↑ TGR5 expression, ↑ GLP-1 (microbiota-dependent BA remodeling)	↓ Hepatic fat (CAP), ↓ TG/FFA, modest glycemic benefits	He et al. (2024)
RCT, n = 58, NAFLD	Phospholipid curcumin 250 mg/day, 8 weeks	↓ CDCA, ↓ TCA, ↓ LCA (NMR metabolomics)	Metabolomic shifts; exploratory clinical signals	Chashmniam et al. (2019)
RCT, n = 70, NAFLD	Curcuminoids 500 mg + piperine 5 mg/day, 12 weeks	Not measured	↓ ALT/AST/ALP, improved ultrasound severity	Panahi et al. (2019)
Randomized comparative, n = 180, NAFLD	Fructus akebiae extract + UDCA vs. UDCA, 250 mg, bid, 24weeks	BA-oriented rationale; endpoints not measured	↑ Clinical efficacy; ↓ ALT/AST/TG/TC; symptom improvement	Hongguang et al. (2019)
RCT,n = 100, presumed NASH + T2D	Berberine ursodeoxycholate 1,000 mg, tid, 18 weeks	BA salt therapy; BA endpoints NR	↓ Liver fat; ↓ liver enzymes/lipids; ↓ weight; ↑ glycemic control	Harrison et al. (2021)

## 7 Future research directions

Recent studies suggest that bile acid metabolism plays a role in the development of NAFLD, particularly through its impact on lipid peroxidation. Lipid peroxidation, especially in its iron-dependent form, is a hallmark of ferroptosis and has been implicated as a key driver in NAFLD progression. Current research on bile acid–induced lipid peroxidation mainly emphasizes ROS generation, with ferroptosis and lipid peroxidation interconnected via the GSH/GSSG system and ROS balance. However, the precise mechanisms by which bile acids promote lipid peroxidation and trigger ferroptosis remain unclear. Future studies should focus on clarifying whether bile acids regulate the GSH/GPX4 pathway and thereby influence ferroptosis in NAFLD, as well as on understanding how ferroptosis mechanisms affect disease progression and prognosis.

## 8 Conclusion

In summary, bile acid metabolism plays a pivotal role in the onset and progression of NAFLD. It regulates hepatic lipogenesis, participates in lipid oxidation, and is closely linked to hepatic steatosis, hepatocyte ballooning, and liver fibrosis. Bile acids modulate ROS, influencing lipid peroxidation, which is connected to ferroptosis via the GSH/GSSG system and ROS. By regulating bile acid metabolism, TCM improves hepatic steatosis, inflammation, and fibrosis, offering a promising therapeutic strategy for NAFLD and underscoring the potential of TCM in treating metabolic diseases. These findings highlight the interplay between bile acid metabolism, lipid peroxidation, and ferroptosis, providing new insights into NAFLD pathophysiology and identifying potential therapeutic targets for clinical treatment, thus contributing to the development of novel therapeutic strategies for metabolic diseases.
